# Radiofrequency thermocoagulation for the treatment of refractory focal status epilepticus

**DOI:** 10.1002/epd2.70091

**Published:** 2025-09-10

**Authors:** Odile Feys, Julia Makhalova, Thomas Manners, José‐David Herrera‐García, Romain Carron, Fabrice Bartolomei

**Affiliations:** ^1^ APHM, Timone Hospital, Epileptology and Cerebral Rhythmology Marseille France; ^2^ Aix‐Marseille Université, INSERM, INS, Institut de Neurosciences Des Systèmes Marseille France; ^3^ Aix Marseille Univ, CNRS, CRMBM Marseille France; ^4^ Epilepsy Unit, Neurology Department Virgen de Las Nieves University Hospital Granada Spain; ^5^ APHM, Timone Hospital, Medico‐Surgical Unit of Epileptology, Functional and Stereotactic Neurosurgery Marseille France

**Keywords:** epileptogenicity markers, radiofrequency thermoablation, SEEG, status epilepticus

## Abstract

This case study reports the first documented use of stereoelectroencephalography (SEEG)‐guided radiofrequency thermocoagulation (RFTC) to treat refractory status epilepticus (RSE). A 33‐year‐old woman with drug‐resistant epilepsy and recurrent RSE underwent SEEG to define her epileptogenic zone. A new RSE started shortly before and continued during the SEEG exploration, being unresponsive to multiple antiseizure medications, vagal nerve stimulation, and corticosteroid therapy. SEEG‐signal quantification based on ictal biomarkers, that is, epileptogenicity index and connectivity epileptogenicity index, identified the epileptogenic zone network (EZN) within the mesial prefrontal, premotor, and parietal cortex, with major implication of the anterior‐middle and posterior cingulate cortex. RFTC was performed on SEEG‐identified targets within the EZN and resulted in rapid cessation of electroclinical seizure activity and full recovery from motor deficits. Seizure frequency remained reduced by over 90% at 4 months post‐procedure. This case highlights the potential of RFTC as a possible therapeutic option for RSE by directly disrupting critical network nodes responsible for seizure generation and propagation. The findings also suggest a broader role of SEEG not only for diagnostic purposes but also for the therapeutic management of refractory seizures, including status epilepticus.

**Key points**

- Radiofrequency thermocoagulation can be used as an alternative treatment of refractory focal status epilepticus.
- A transient increase in the interictal spike rate does not mean failure of radiofrequency thermocoagulation.
- The effect of radiofrequency thermocoagulation could be explained by the disruption of the status epilepticus network.

## 1 INTRODUCTION

Status epilepticus (SE) occurs when the mechanisms of seizure termination fail or mechanisms that lead to abnormally prolonged seizures are initiated. Refractory status epilepticus (RSE) is defined after failure of at least two intravenous antiseizure medications (ASMs)^1^ and occurs in 30–55% of SE.^2^, ^3^ Alternative therapeutics (resective/disconnective surgery or neuromodulation) can then be used.^4^ Stereoelectroencephalography (SEEG) recordings allow for precise estimation of the extent of the brain networks involved in the SE.^5^

SEEG‐guided radiofrequency thermocoagulation (RFTC) may be efficient in improving seizure control in patients with drug‐resistant epilepsy (DRE) non‐eligible for resective surgery.^6^ Herein, we describe the first application of SEEG‐guided RFTC to treat RSE.

## 2 METHODS

A 33‐year‐old woman with DRE, with a history of febrile seizures and tonic seizures of the right hemibody during infancy, underwent a presurgical evaluation. Epilepsy diagnosis followed the first tonic–clonic seizure evolving to RSE at 22 years, then weekly to daily focal seizures and 3–4 focal to bilateral tonic–clonic seizures per year occurred. Her initial seizure semiology was characterized by blinking, either with right head/gaze deviation, or with tonic posture of the right arm, and arm and leg myoclonus, loss of awareness and falls. At time of evaluation, the ictal semiology was modified and comprised subjective “loss of right hand” sensation followed by right arm dystonic/myoclonic movements, or right arm paresis, with partial or no alteration of awareness. Recurrent SE and RSE, mainly induced by the reduction of Clonazepam dosage due to the side effects, required multiple prolonged intensive care unit stays.

The noninvasive presurgical assessment comprised (i) a video‐EEG showing a left hemispheric background slowing, left frontal and fronto‐central median interictal spikes, focal seizures with the above‐described semiology, or with behavioral arrest, head and eyes version to the left and atonic head drop, both types being associated with a fronto‐centro‐parietal median then bilateral, left predominant, rhythmic sharp‐wave discharge, (ii) a brain MRI showing a left hemispheric atrophy, (iii) a brain fluorodeoxyglucose‐positron emission tomography showing a left hemispheric hypometabolism, and (iv) a neuropsychological assessment showing a low overall cognitive efficiency predominant in nonverbal/executive functions. Auto‐immune/Rasmussen encephalitis was excluded.

SEEG was indicated to define the epileptogenic zone network (EZN, i.e., a hierarchical organization of the brain region producing seizures versus propagating seizures (PZN)^7^, ^8^) with hypotheses in the left motor‐premotor or parieto‐premotor system. At the time of the SEEG, the patient was under five ASMs (Lamotrigine, Levetiracetam, Carbamazepine, Clonazepam, Phenobarbital) and an active vagal nerve stimulator. Seizure frequency increased to multiple daily seizures a few months before the SEEG implantation.

**FIGURE 1:**
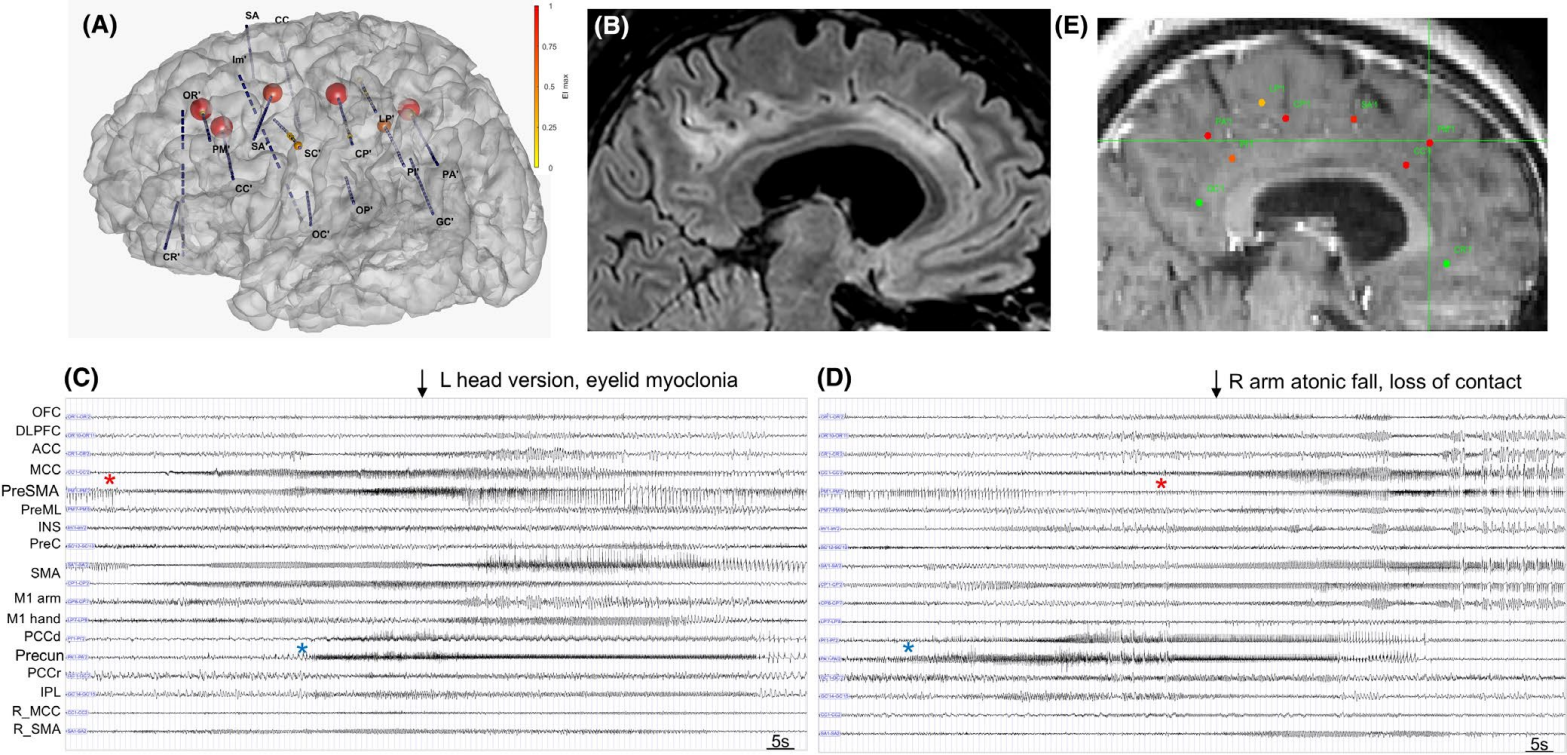
Ictal SEEG, MRI, and epileptogenicity quantification in a patient with refractory status epilepticus. (A) Tridimensional representation of the SEEG implantation on the patient's 3d brain mesh: 14 left hemispheric electrodes sampled the mesial and lateral prefrontal (CR′, OR′, PM′, CC′), orbitofrontal (OR′), mesial and lateral premotor (SA′, SC′, Im′), insulo‐opercular (Im′, OC′, OP′), primary motor (CP′, LP′), mesial, and lateral parietal (PI′, GC′, PA′) regions. Two right electrodes sampled the right mesial and lateral premotor regions (CC, SA, not shown). Quantified ictal epileptogenicity markers (the maximal normalized epileptogenicity index (EI) values) are represented by the color (yellow to red) and size of the spheres on the respective electrode contacts. (B) A sagittal T2‐weighted FLAIR MRI image of the left hemisphere showing cortical hyperintensity within the anterior, middle, posterior cingulate cortex and the precuneus. (C, D) SEEG recordings of subintrant electroclinical seizures involving the mesial wall of the left hemisphere. (C) Low‐voltage fast discharges started within the anterior aspect of the left middle cingulate cortex (CC′1–2) and the adjacent medial prefrontal cortex (PM′1–2, red asterisk), with rapid involvement of the mesial premotor (supplementary motor area, SA′1–2) cortex, followed 30 s later by a fast discharge within the left posterior cingulate cortex (PI′1–2) cortex and the precuneus (PA′1–2, blue asterisk), with secondary involvement of the left primary motor cortex (LP′7–8). Clinically, head version to the left and eye lid myoclonia with alteration of awareness are observed concomitant to a vast discharge involving the left mesial structures in the central phase of the seizure. (D) Low‐voltage fast discharge starting within the left precuneus and posterior cingulate cortex (blue asterisk), involving the primary motor cortex and then followed by a fast discharge within the middle cingulate cortex and the mesial prefrontal cortex (red asterisk), implicating the ipsilateral supplementary motor area. Clinically, atonic fall of the right arm and loss of consciousness are observed. (E) The maximal EI values represented according to a color scale from yellow to red on the respective SEEG electrode contacts within a sagittal T1‐weighted MRI image of the left hemisphere. The contacts CC′1–2 (left middle cingulate cortex), PM′1–2 (left mesial prefrontal), SA′1–2 (left supplementary motor area), CP′1–2 (posterior aspect of the left supplementary motor area), PI′1–2 (left posterior cingulate cortex), and PA′1–2 (left precuneus) showed high EI values and belonged to the epileptogenic zone network.

Sixteen electrodes (14 left and 2 right, 10–18 contacts, length 2 mm, diameter .8 mm, 1.5 mm apart; Alcis, France) were implanted stereotactically with ROSA® robotized assistance (Zimmerbiomet, US) and intra‐operative CT control (Moebus, Airo, Strycker) (Figure 1A), and control with post‐implantation CT and MRI. SEEG was recorded on a 256‐channel Natus system, sampled at 1024 Hz, with high‐pass filter (1 Hz) and antialiasing low‐pass filter (340 Hz). The automatic localization was performed with GARDEL software (https://meg.univ‐amu.fr/wiki/GARDEL:presentation).^9^

The study was approved by the APHM Institutional Board and informed consent was obtained.

Signal analyses of SEEG recordings (before/after intravenous clonazepam, before/after RFTC and 24 hours after RFTC) were performed in a bipolar montage using the AnyWave software (https://meg.univ‐amu.fr/wiki/AnyWave). Interictal spikes were detected automatically using Delphos software.^10^ In the context of this study, we defined the epileptogenic zone as a potential network of highly epileptogenic structures (“epileptogenic zone network”), quantitatively defined by epileptogenicity indices, etc.^7^ For this purpose, we used Epileptogenicity Index (EI)^11^ and connectivity Epileptogenicity Index (cEI),^12^ performed to evaluate epileptogenicity of sampled brain structures and identify the regions involved in the EZN (EI ≥ .40 and/or cEI ≥ .65, displayed on the patient's brain using 3DViewer^9^).

The RFTC (LG2‐sEEG lesion generator, Inomed, Germany) was performed at the patient's bed under SEEG monitoring; the French guidelines of standard care, with respect to critical vasculature and primary motor cortex.

## 3 RESULTS

At the time of the SEEG recordings, the patient experienced a focal SE with subintrant usual seizures and motor seizures with negative myoclonic movements of the right upper limb, without return to the baseline state. In particular, there was a persisting Todd paresis of the right upper/lower limbs that did not correspond to the patient's habitual clinical presentation documented during the phase I presurgical assessment (Figure S1). A pre‐implantation T2‐weighted MRI showed cortical hyperintensity within the left anterior/middle/posterior cingulate cortex and the precuneus (Figure 1B). RSE was diagnosed after the failure of intravenous Clonazepam and Lacosamide. No improvement occurred following methylprednisolone pulses.

Video‐SEEG recording showed ~30/h subintrant seizures, alternating with < 2 min interictal periods. Consecutive/alternative dynamic involvement of the left medial frontal (the middle cingulate cortex, CC′1–2, and mesial prefrontal cortex, PM′1–2) and medial parietal (the posterior cingulate cortex, PI′1–2, and precuneus, PA′1–3) regions characterized the seizures, with rapid involvement of the mesial premotor (supplementary motor area, SA′1–2) and of the primary motor cortex (Figure 1C,D). EI and cEI showed high epileptogenicity within the above‐mentioned medial prefrontal cortex, supplementary motor area, and parietal structures, while the lateral premotor and motor cortex showed high cEI values only (Figure 1A,E).

**FIGURE 2:**
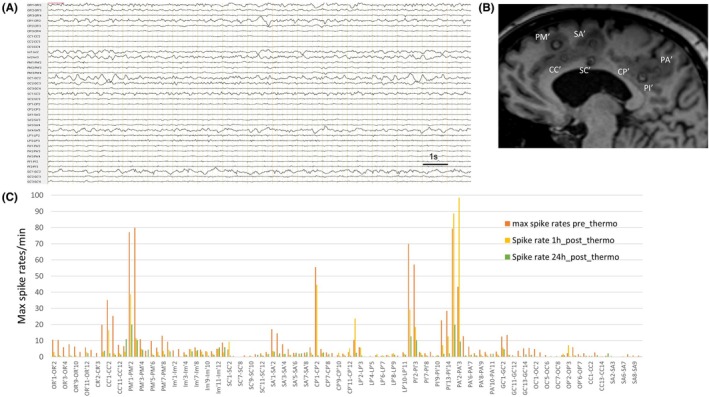
SEEG findings and spike rates dynamics after radiofrequency thermocoagulation for refractory status epilepticus. (A) Interictal awake SEEG recording 24 h following RFTC showing cessation of subintrant seizures and interictal spikes, and signal hypovoltage over the RFTC targets. (B) A sagittal T1‐weighted MRI image of the left hemisphere 48 h post‐RFTC showing thermolesions within the left mesial prefrontal, the supplementary motor area, the middle and posterior cingulate cortex and the precuneus. (C) Graph showing the maximal spike rates/min quantified for each SEEG contact before RFTC, 1 h and 24 h post‐RFTC.

CC′1–3, PM′1–3, SA′1–3, CP′1–2, PI′1–3, and PA′1–3 (i.e., left medial prefrontal cortex, supplementary motor area, middle cingulate cortex, posterior cingulate cortex, and precuneus) were determined as targets for RFTC (Figure 2B). RSE ceased within 24 h following RFTC, with cessation of subintrant electrographic seizure activity (Figure 2A), arrest of electroclinical and significant reduction of infraclinical seizures, rapid recovery of the right arm deficit, and progressive recovery of the right leg paresis. At 4 months post‐RFTC, the seizure frequency was reduced by more than 90%. The recuperation of the right hemiparesis was complete.

## 4 DISCUSSION

To the best of our knowledge, this is the first description of RFTC treatment of RSE. This case is particularly illustrative of two aspects: the first is the direct observation of the antiseizure effect of thermocoagulation and the second is related to its use in SE. In our case, SEEG had been planned before the onset of SE. Few cases of RSE have been studied with SEEG, demonstrating continuous ictal changes resolved by surgery.^5^ In our case, the SE remained primarily confined to the medial aspect of the left hemisphere, particularly the cingulate cortex, with subintrant seizures alternating between the anterior and posterior cingulate cortex. Due to the deep location of the seizure source, scalp EEG may be utterly blind to pathological cingulate activity,^13^ resulting in the risk of delayed diagnosis of SE.

While benzodiazepines and ASMs remain the first‐line treatment for SE,^2^ RFTC could be considered as an alternative treatment in cases of recurrent SE. This alternative treatment could be rapidly utilized in the management of RSE to avoid neuronal death and brain damage.

RFTC is used to destroy or disconnect brain areas by applying a high‐frequency alternating current to coagulate brain tissue.^14^ In our case, the effect of RFTC could be explained by the disruption of the network responsible for generating/propagating SE.^15^ By eliminating some key EZN nodes, RFTC hinders the generation and propagation of SE through the disruption of large‐scale brain networks,^16^, ^17^ similarly to previous work in patients with large non‐resectable epileptogenic networks such as described in Lennox–Gastaut syndrome.^18^ RFTC in this indication also opens the way for other minimally invasive curative approaches such as laser interstitial thermal therapy.^19^

In our patient, RFTC did not result in seizure freedom due to the limited volume of thermocoagulation compared with the extensive epileptogenic network involving the entire medial wall of the left hemisphere. A full resective surgery or alternative complete ablation strategy would carry a high risk of postoperative functional deficit; whereas the patient reports a highly favorable outcome in terms of seizure frequency.

A transient increase in the interictal spike rate was observed in the mesial parietal EZ nodes, while there was a prompt decrease in the spike rates within the prefrontal and premotor nodes, 1 h post‐RFTC. A high variability of spike rates after RFTC was already described.^20^ We hypothesize that this short‐term increase in the spiking rate highlights the propagated spikes from non‐coagulated to coagulated areas due to the arrest of intrinsic epileptic activity in the latter sites. It was shown that interictal activity significantly decreases only within the EZN and thermocoagulated areas, but not in distant regions. In these distant zones, activity changes variably, with occasional increases in spike rates.^21^ Moreover, a recent study described increased connections from non‐coagulated to coagulated areas after RFTC.^22^ Nonetheless, the spike rates may decrease later after RFTC due to a running‐down phenomenon,^23^, ^24^ which was also observed for all the RFTC targets in the present case.

Finally, this case demonstrates that SEEG can be performed in the frame of RSE with a therapeutic intent. Indeed, RFTC enables disruption of the involved network even in patients who cannot benefit from resective surgery to tackle RSE.

## FUNDING INFORMATION

No funding to report.

## CONFLICT OF INTEREST STATEMENT

None of the authors has any conflict of interest to disclose.

## PATIENT CONSENT

The study was approved by the APHM Institutional Board, and written informed consent was obtained.

**Test yourself**

1. What type of status epilepticus does the patient have?
  - Generalized status epilepticus
  - Pharmacosensitive status epilepticus
  - Refractory status epilepticus
  - New‐onset refractory status epilepticus
  - This is not a status epilepticus
2. What treatment has proven effective?
  - Radiofrequency thermocoagulation
  - Lacosamide IV
  - Vagal nerve stimulation
  - Transcranial direct current stimulation
  - Clonazepam IV
3. By what mechanism was the treatment effective?
  - It blocks sodium channels
  - It interrupts the status epilepticus genesis and propagation network
  - It removes the epileptogenic zone
  - It potentiates the antiseizure medications effect
  - It stimulates the vagal nerve

*Answers may be found in the* *supporting information*

## Supporting information


Figure S1.

Data S1.

## Data Availability

The data that support the findings of this study are available from the corresponding author upon reasonable request.
