# Supplementary material for: Radiofrequency thermocoagulation for the treatment of refractory focal status epilepticus
Source: Epileptic Disord. 2025 Sep 10;27(6):1292–7. doi: 10.1002/epd2.70091 (PMC12747700; doi:10.1002/epd2.70091)
Supplement: Supplementary file 1 — Figure S1. [file EPD2-27-1292-s002.docx]

**Supplemental Figure 1. Presurgical scalp EEG and MRI.**

**A.** 20 seconds of ictal scalp EEG signal recorded according to the 10-20 montage, including temporal basal electrodes, and displayed in longitudinal bipolar montage. **B.** A sagittal T2 FLAIR MRI image of the left hemisphere at the time of the first presurgical assessment, four years before the SEEG implantation. **C.** A sagittal T2 FLAIR MRI image of the left hemisphere acquired for neuronavigation planification just prior to the SEEG implantation, showing cortical hyperintensity within the anterior, middle, posterior cingulate cortex and the precuneus. **D.** 20 seconds of interictal scalp EEG signal (same montage as A.).
