# Supplementary material for: Radiofrequency thermocoagulation for the treatment of refractory focal status epilepticus
Source: Epileptic Disord. 2025 Sep 10;27(6):1292–7. doi: 10.1002/epd2.70091 (PMC12747700; doi:10.1002/epd2.70091)
Supplement: Supplementary file 2 — Data S1. [file EPD2-27-1292-s001.docx]

Answers

1. C

2. A

3. B
